# Long-term results of postoperative unsuspected small cell lung cancer on real-world data

**DOI:** 10.1186/s12885-022-10341-9

**Published:** 2022-12-02

**Authors:** Juntang Guo, Leilei Shen, Zhipeng Ren, Yang Liu, Chaoyang Liang

**Affiliations:** 1 Department of Thoracic Surgery, The First Medical Center, The Chinese PLA General Hospital, Beijing, People’s Republic of China; 2 Department of Thoracic Surgery, The Hainan Hospital, The Chinese PLA General Hospital, Sanya, Hainan Province People’s Republic of China

**Keywords:** Lung cancer, SCLC, Surgery, Overall survival rate

## Abstract

**Background:**

In traditional opinion, solid pulmonary nodule suspected lung cancer should be confirmed by pathology before the operation to exclude small cell lung cancer (SCLC), considering SCLC tends to be aggressive and surgical effect in the management of SCLC remains controversial. The aim of this study was to evaluate the survival result and risk factors of postoperative unsuspected SCLC.

**Methods:**

A total of 120 patients with postoperative unsuspected SCLC who were confirmed by pathology and referred to Chinese PLA General Hospital between 2000 and 2021 were retrospectively analyzed (surgery group). Additionally, 120 patients with limited-stage SCLC who underwent chemotherapy and radiotherapy in the same period were enrolled in the chemoradiotherapy group.. Kaplan–Meier method was used to estimate survival; the Log-Rank test was used to compare survival rates between different groups; a COX stepwise regression model was used for multivariate analysis.

**Results:**

Among 120 patients in the surgery group, 28 were with central type and other 92 with peripheral type. The median survival (OS) was 44.85 months, and the 5-year survival rate was 46%. The 5-year survival rates for stage I, II, and III were 52.1%, 45.4%, and 27.8%, respectively. The mean disease-free survival time (DFS) was 30.63 ± 4.38 months, and the 5-year DFS rate was 31.5%. In the chemoradiotherapy group, the mean OS was 21.4 ± 4.26 months, and the 5-year survival rate was 28.3%. The 5-year survival rates for clinical stage I, II, and III were 42.5%, 39.8%, and 20.5%, respectively. The mean progression-free survival (PFS) was 10.63 ± 3.6 months. In the surgery group, one-way ANOVA revealed that the gender, symptoms, smoking history, tumor location, and postoperative radiotherapy were not associated with OS (*P* ≥ 0.05), while age, surgical approach, surgical method, N stage, TNM stage, and vascular tumor thrombus were related to OS (*P* < 0.05). Multivariate analysis indicated that the N stage was associated with OS (HR = 1.86 *P* = 0.042).

**Conclusion:**

Surgery and adjuvant therapy were found to have encouraging outcomes in postoperative unsuspected SCLC. Patients with stage I, stage II and part of stage IIIA SCLC could benefit from surgery and the standard lobectomy, and systematic lymph node dissection, is also recommended for these patients.

## Introduction

Small cell lung cancer (SCLC) is characterized by a high degree of malignancy, rapid progression, and poor prognosis. It is also often accompanied by mediastinal lymph node metastasis or distant metastasis at the time of diagnosis. Hence, SCLC was previously recognized as a systemic disease. Chemotherapy and radiotherapy are the standard treatments for this condition, while surgery has not been acknowledged as a routine procedure [1, 2]. However, with the popularization of chest computed tomography (CT) examinations, an increasing number of lung cancers are being detected earlier than ever before. In real-world clinical practice, some patients have postoperative pathologically confirmed SCLC, known as postoperative unsuspected SCLC. Recent large-cohort studies investigating the role of surgery in SCLC patients demonstrated that surgery could provide a significant survival benefit for patients with a broader range of disease stages (I–IIIA) [3–5]. Nevertheless, the survival outcome and related risk factors of postoperative unsuspected SCLC remain unclear.

This study aimed to retrospectively analyze the patients with no preoperative pathological diagnosis and postoperative pathologically confirmed SCLC, their clinical characteristics, and imaging manifestations to explore the survival and risk factors that could guide the preoperative differential diagnosis of SCLC in the future.

## Materials and methods

### Research objects

Patients who underwent surgical treatment in the PLA General Hospital between January 2000 and June 2021 and were confirmed by pathology with postoperative unsuspected SCLC were included in the study. Inclusion criteria were the following: no severe complications during the perioperative period; survival time > 3 months after the operation. The exclusion criteria referred to patients with preoperative pathologically confirmed SCLC who received neoadjuvant chemotherapy.

A total of 120 cases were included, and their general information and clinical data were collected. Additionally, 467 patients with limited SCLC who underwent 1st-line systemic chemotherapy and radiotherapy in our hospital between January 2000 and December 2020 were enrolled in the chemoradiotherapy group. Propensity score matching (PSM) was performed between the surgery and chemoradiotherapy groups in a 1:1 manner. Age, sex, tumor size on CT, and clinical N stage were included in PSM (Table 1).

**Table 1:** Patient characteristics of the surgery group versus the chemoradiotherapy group after propensity score–matched

	**Surgery (*n* = 120)**	**Chemoradiotherapy (*n* = 120)**	***P*-value**
Age (years), mean (SD)	60.2 (9.4)	61 (8.6)	0.83
Gender, no. (%)			0.56
Male	93 (77.5)	89 (74.2)	
Female	27 (22.5)	31 (25.8)	
Smoking history, no.(%)			0.938
Yes	79 (65.8)	82 (68.3)	
None	41 (34.2)	38 (31.8)	
Tumor location, no. (%)			0.76
RUL	28 (23.3)	27 (22.5)	
RML	15 (12.5)	14 (11.7)	
RLL	25 (20.8)	24 (20)	
LUL	28 (23.3)	31 (25.8)	
LLL	24 (20)	24 (20)	
Tumor size(cm), mean(SD)	3.6 (1.97)	3.5 (2.05)	0.96

### Preoperative examination

Distant metastases were excluded based on the following examinations: routine preoperative brain magnetic resonance imaging (MRI) or CT scan, chest CT scan, enhanced chest CT scan, whole-body bone scan, ultrasound of liver, gallbladder, pancreas, spleen, adrenals, and neck and supraclavicular lymph node ultrasound. Patients with pulmonary nodules located in the hilum underwent bronchoscopy. After 2008, some patients underwent whole-body positron emission tomography (PET)-CT examination. All patients were tested for serum tumor markers, including carcinoembryonic antigen (CEA), neuron-specific enolase (NSE), cytokeratin fragment 19 (CYFRA21-1), squamous cell carcinoma-associated antigen (SCC), etc.

### Treatment methods

All 120 patients in the surgery group underwent surgical treatment, including posterolateral thoracotomy and thoracoscopic approach. Surgical methods included wedge resection, segmentectomy, lobectomy, double lobectomy, sleeve lobectomy, and pneumonectomy. Except for mediastinal lymph node sampling in wedge resection, hilar and mediastinal lymph node dissection was performed in all other procedures. Pathological staging was done based on the 7^th^ edition of AJCC’s tumor, node, and metastases (TNM) staging criteria [6].

All patients received systemic chemotherapy after surgery, with the most commonly used chemotherapy regimens including EP (etoposide/cisplatin), EC (etoposide/carboplatin), or IP (irinotecan hydrochloride/cisplatin). Some patients received postoperative thoracic radiotherapy and prophylactic whole-brain radiotherapy. Eight patients received chemotherapy combined with immunotherapy, and 3 patients received chemotherapy combined with vascular-targeted therapy.

All 120 patients in the chemoradiotherapy group received chemotherapy as the first-line treatment. Irradiation of the primary tumor and mediastinal lymph nodes was performed following 4 cycles of chemotherapy. The mean radiation dose was 54 Gy (30-72 Gy). After radiotherapy, 2–4 cycles of chemotherapy were administered according to patient’s tolerance. Six patients received immunotherapy combined with chemotherapy.

### Follow-up

The patients underwent regular outpatient re-examination after surgery every 3 months within 2 years after surgery and every 6 months thereafter. The re-examinations included brain MRI or CT scan, chest CT scan, whole-body bone scan, ultrasound of liver, gallbladder, pancreas, and spleen + adrenal gland, ultrasound of the neck and supraclavicular lymph nodes, and serum tumor markers. Besides outpatient follow-up, all patients were followed up by telephone. The last follow-up time was September 16, 2021.

The study's primary endpoint was overall survival (OS), which was defined from the date of surgery to the date of death from any cause or the date of the last follow-up. Progression-free survival (PFS) was defined as the time from the first therapy dose to disease progression or death, whichever occurred first. Disease-free survival (DFS) was defined as the time from surgical tumor resection until disease recurrence, which also included patients without relapse who were censored on the date of the last follow-up or death.

### Statistical analysis

Stata software (Version 15 StataCorp, TX, USA) was used for all statistical analyses. Continuous variables and dichotomous variables were compared with t-test and Chi-square test, respectively. Survival was estimated using the Kaplan–Meier method. The survival rate comparison between different groups was performed with the Log-Rank test. A COX stepwise regression model was used for multivariate analysis. A *p*-value < 0.05 was considered statistically significant.

## Results

### General characteristics

There were 8238 cases of lung cancer confirmed by pathology after surgery, including 8112 cases of non-SCLC and 126 cases of SCLC. SCLC accounted for 1.53% of lung cancers managed by surgical resection. As 4 patients who received neoadjuvant therapy and 2 patients who lacked follow-up data were excluded, 120 patients were finally included in the study as the surgery group. Among these 120 patients, 93 were men and 27 were women, aged 35–80 years old, with an average age of 60.25 ± 9.38 years. Respiratory symptoms such as cough and blood in sputum were found in 48 patients, while the remaining 72 were asymptomatic and were incidentally diagnosed during physical examination. There were 79 patients who were smokers, 64 of whom were heavy smokers (smoking index > 600 cigarettes), while the remaining 41 patients had no smoking history. With reference to co-morbidities, 1 patient had a history of squamous cell carcinoma of the lung, 2 had a history of thyroid cancer, and 16 had a family history of lung cancer.

Chest CT showed a central mass in 28 (23%) cases and peripheral mass in the remaining 92 (77%) cases, while there were only 2 cases with non-solid nodules. Six patients were found with accompanying nodules in the ipsilateral lung. According to the CT images, 107 (89%) cases were accompanied by lobulation, 13 (11%) cases were with no lobulation, 45 (38%) cases had smooth nodule edges, and 75 (62%) cases had blurred edges. Only 5 cases had a vacuolar sign. Mediastinal lymphadenopathy was found in 44 (37%) cases, while 78 (63%) had no obvious lymphadenopathy. Whole-body PET-CT examination was administered in 38 cases, with an average standardized uptake value (SUV) value of 8.8 ± 4.1.

### Surgical and pathological staging

In the surgery group, 52 (43%) patients underwent thoracotomy and 68 (57%) patients underwent minimally invasive thoracoscopic surgery. Among them, 87 (71.3%) patients underwent lobectomy, 5 underwent pneumonectomy, 6 underwent bilobectomy, and the remaining 14 underwent a sublobar resection. There were no perioperative deaths. R0 resection was achieved in 117 cases, and R1 resection was achieved in 3 cases. Pathological results showed that 109 cases were pure small cell carcinoma, 11 cases were mixed type, 4 cases were combined with large cell carcinoma, and 7 cases were combined with squamous cell carcinoma. According to the largest diameter, the tumor size was 0.8-12 cm, and the average size was 3.61 ± 1.97 cm. In addition, there were 30 cases associated with pleural invasion, 4 cases with tumor spread in the trachea, and 21 cases with intravascular tumor thrombus. Lymph node dissection was performed in 115 cases, and the number of dissected lymph nodes was 1–50, with an average of 11.9 ± 0.69. There were 61 cases of lymph node metastasis, including 40 cases of single-site metastasis and 21 cases of multi-site metastasis.

Pathological staging was as follows: 29 cases in stage IA, 10 cases in stage IB, 15 cases in stage IIA, 22 cases in stage IIB, 37 cases in stage IIIA, 5 cases in stage IIIB, and 2 cases in stage IV. In stage IV, 2 cases were found to have pleural nodules during operation, and intraoperative frozen pathology confirmed metastasis. Immunohistochemistry showed that the average Ki-67 was 75.5 ± 13.2%. There was no significant difference in pathology characteristics between the two subgroups of patients with and without a smoking history in the surgery group (Table 2).

**Table 2:** Comparison of characteristics of pathology for a subgroup of patients with and without smoking history in the surgery group

	**Patients with a smoking history (*n* = 41)**	**Patients without a smoking history (*n* = 79)**	***P-*value**
TNM Stage			0.775
I	14	28	
II	11	23	
III	15	27	
IV	1	1	
Vascular tumor thrombus			0.679
Yes	8	13	
None	33	66	
Ki-67			0.405
≧50	38	78	
< 50	3	1	

### Adjuvant therapy

All 120 patients in the surgery group received 2–13 cycles of systemic chemotherapy, 44 patients received radiotherapy (11 patients in stage I, 6 patients in stage II, and 27 patients in stage III), 17 patients received prophylactic whole-brain radiotherapy, and 8 patients received thoracic radiotherapy. In addition, combination therapy of cytotoxic chemotherapy and programmed death and its ligand 1 (PD-1/PD-L1) antibody immunotherapy were used in 8 patients.

### Survival analysis

**Fig. 1:**
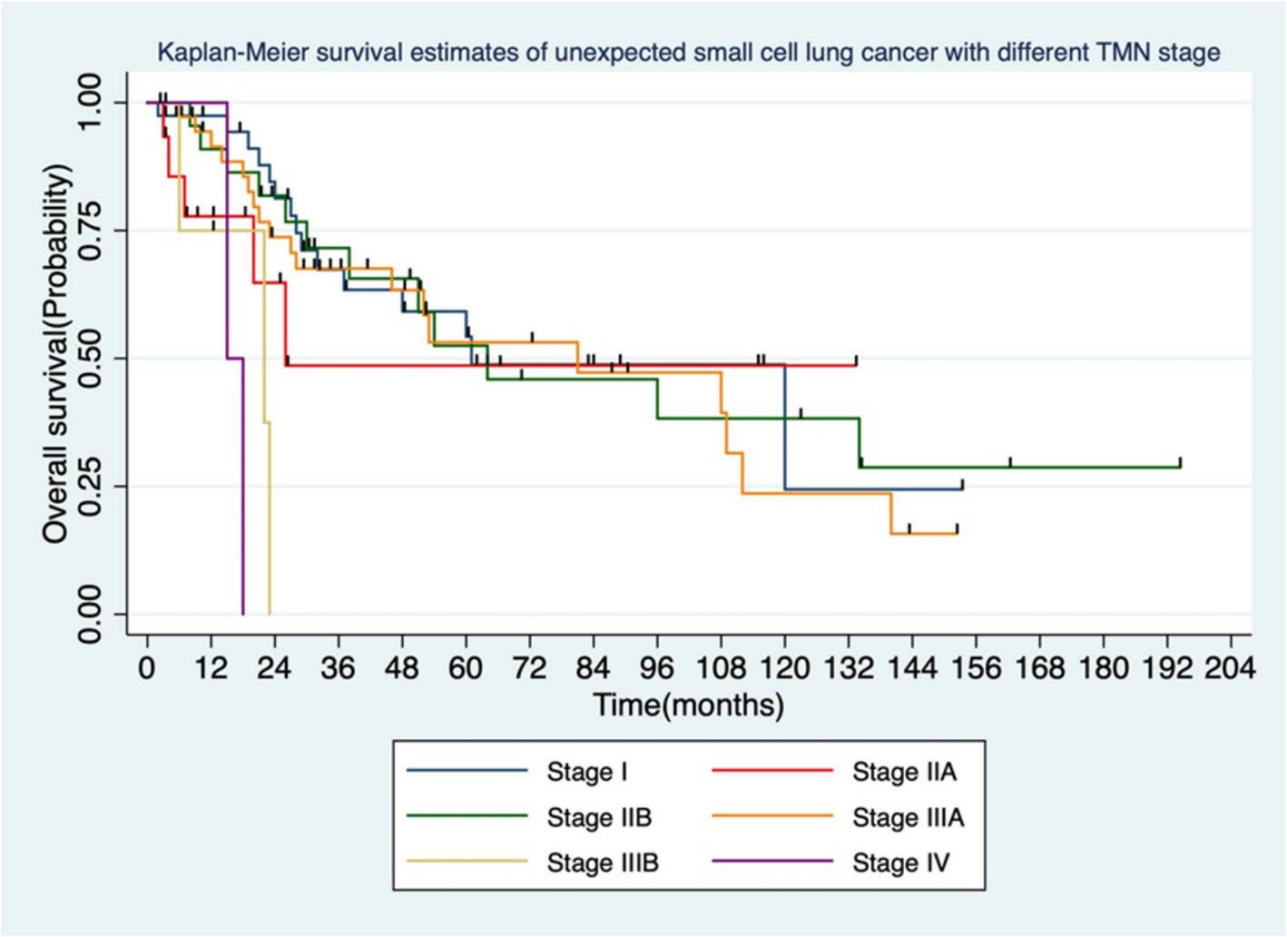
Comparison of survival analysis of different pathological TNM stages

There were no perioperative deaths in the surgery group. The mean follow-up was 51 months, and 48 patients were still alive at the end of the follow-up. The mean overall survival time (OS) was 44.85 ± 3.85 months, and the 5-year survival rate was 46%. The 5-year survival rates for stages I, II, and IIIA were 52.1%, 45.4%, and 27.8%, respectively (Fig. 1). The mean disease-free survival time (DFS) was 30.63 ± 4.38 months, and the 5-year DFS rate was 31.5%.

**Fig. 2:**
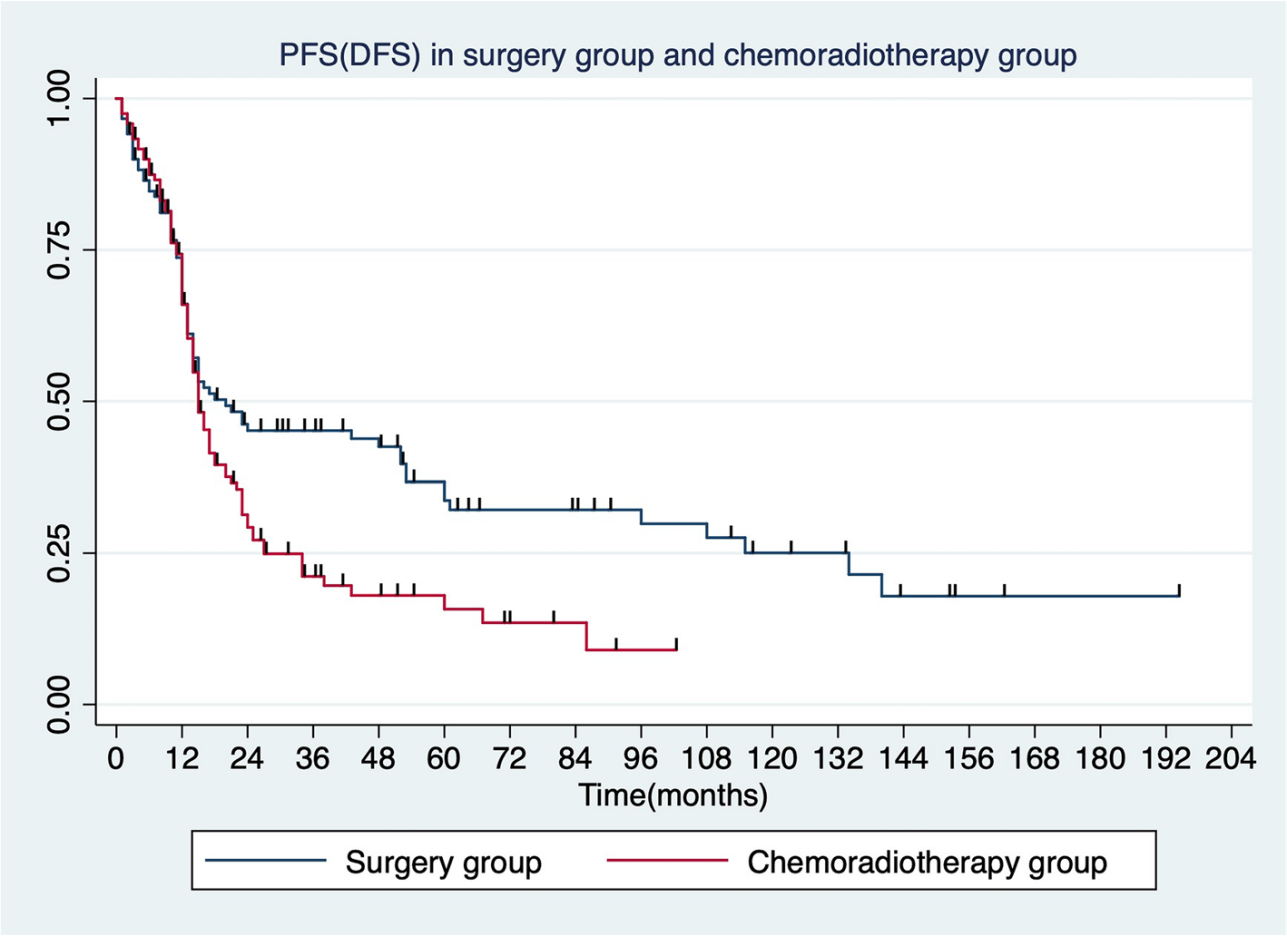
Comparison of PFS (DFS) in the surgery group and the chemoradiotherapy group

In the chemoradiotherapy group, the mean OS was 21.4 ± 4.26 months, and the 5-year survival rate was 28.3%. The 5-year survival rates for clinical stage I, II, and III were 42.5%, 39.8%, and 20.5%, respectively. The mean PFS was 10.63 ± 3.6 months (Fig. 2).

**Fig. 3:**
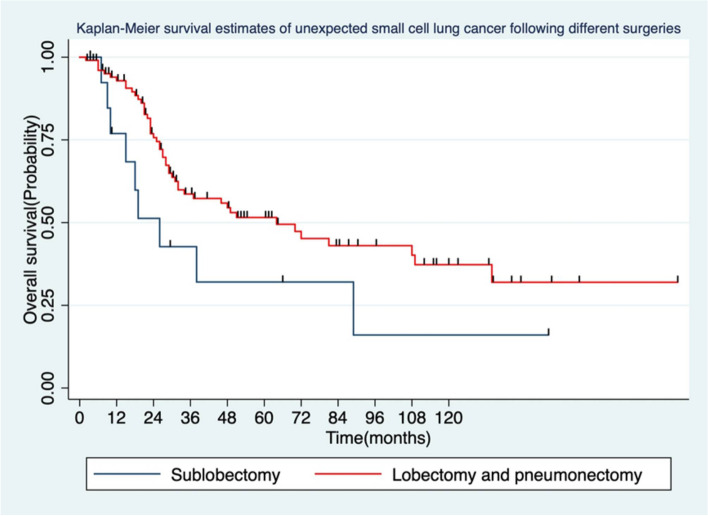
Comparison of survival analysis between lobectomy (including bilobectomy) and other procedures

**Fig. 4:**
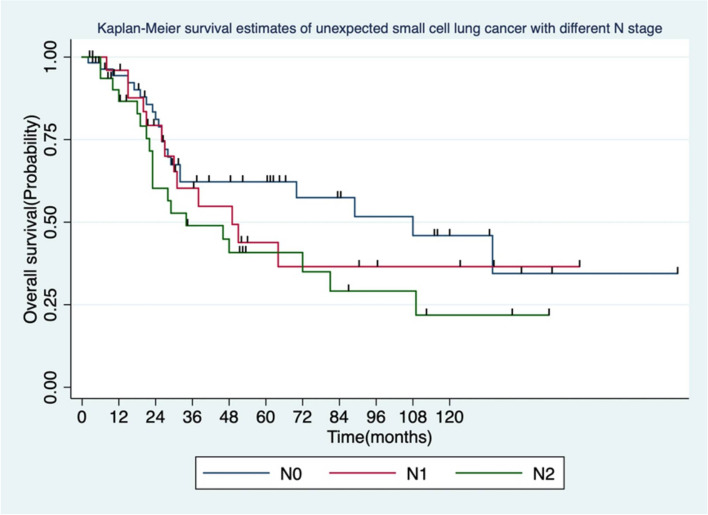
Comparison of survival analysis of different N stages

Further analysis showed that gender, symptoms, smoking history, tumor location, and postoperative radiotherapy were not significantly associated with OS (P ≥ 0.05), while age, surgical approach, surgical method, N stage, TNM stage, and vascular tumor thrombus were related to OS of patients (*P* < 0.05, Table 3, Fig. 3, and Fig. 4). Earlier N and TNM stage were associated with improved DFS compared to the later stage. Lobectomy has no significant DFS benefit compared to other resections.

**Table 3:** Summary of DFS and OS in patients with unexpected small cell lung cancer

Clinical characters	Number (%)	*HR* (95%CI)	Median DFS	*P*-value	*HR* (95%CI)	Median OS	*P*-value
Age(years)				0.083			**0.006**
≥ 60	69 (57.5)	1.53 (0,95–2.47)	16		2.19 (1.24–3.86)	32	
< 60	51 (42.5)	1	52		1	52	
Gender				0.94			**0.718**
Male	93 (77.5)	0.98 (0.57–1.69)	23		1.13 (0.58–2.18)	64	
Female	27 (22.5)	1	15		1	51	
Symptom				0.814			**0.436**
Yes	48 (40.0)	0.95 (0.59–1.51)	18		0.81 (0.48–1.38)	70	
None	72 (60.0)	1	20		1	49	
Smoking history				0.697			**0.656**
Yes	79 (65.8)	0.91 (0.57–1.46)	23		0.88 (0.51–1.52)	64	
None	4 1(34.2)	1	15		1	46	
Tumor location				0.806			**0.945**
Central lesion	28 (23.3)	1.07 (0.63–1.80)	17		1.02 (0.56–1.84)	64	
Peripheral lesion	92 (76.7)	1	21		1	48	
Surgical approach				0.253			**0.003**
Open	52 (43.3)	0.75 (0.46–1.22)	48		0.43 (0.25–0.75)	108	
VATS	68 (56.7)	1	15		1	29	
Type of operation				0.065			**0.016**
Lobectomy and pneumonectomy	106(88.3)	0.53 (0.27–1.03)	23		0.51 (0.25–1.04)	64	
Sublobectomy	14(11.7)	1	7		1	26	
Number of lymph nodes dissected				0.390			**0.576**
≧15	39 (32.5)	0.80 (0.48–1.33)	52		0.84 (0.47–1.53)	64	
< 15	81 (67.5)	1	16		1	49	
N staging				0.046			**0.030**
N0	58(50)	1	48		1	108	
N1	26(22.4)	1.08 (0.58–2.02)	43		1.21 (0.65–2.58)	49	
N2	32(27.6)	1.72 (0.99–2.97)	13		1.75 (0.94–3.25)	34	
TNM staging				0.027			**0.021**
Stage I	42 (35)	0.45 (0.23–1.86)	60		0.45 (0.23–1.86)	89	
Stage II and IIIA	78 (65)	1	15		1	51	
Vascular tumor thrombus				0.583			**0.000**
Yes	21 (17.5)	0.83 (0.42–1.62)	23		3.12 (1.59–4.25)	38	
None	99 (82.5)	1	20		1	64	
Histology				0.717			**0.493**
Pure SCLC	109 (90.8)	1.18 (0.47–2.94)	18		1.5 (0.47–4.8)	49	
Mixed SCLC	11 (9.2)	1	61		1	55	
Postoperative radiotherapy				0.619			**0.598**
Yes	42 (34.5)	0.88 (0.55–1.43)	23		1.15 (0.68–1.97)	29	
None	78 (65.5)	1	18		1	64

Multivariate analysis showed that the N stage was associated with OS (HR = 1.86 *P* = 0.042), unlike tumor location and surgical method (Table 4).

**Table 4:** Multivariate analysis of the risk factors affecting the survival of unsuspected small cell lung cancer after surgery

Factors	*HR*	95%CI	*P*-value
Type of operation			
Sublobectomy	1		
Lobectomy and pneumonectomy	O.73	0.30–1.75	0.485
N staging			
N0	1		
N1 and N2	1.42	0.97–2.81	0.042
Number of lymph nodes dissected			
≧15	1		
< 15	0.93	0.49–1.70	0.791

## Discussion

SCLC is a highly malignant tumor characterized by rapid progression. The median survival time of affected patients is 17 months, while a 5-year survival rate for patients with the limited-stage disease is only 10% [7]. At present, the treatment of SCLC is mainly based on chemoradiotherapy, while the role of surgical treatment remains controversial. In the last century, two prospective trials reported that surgery was not beneficial for SCLC [1, 2]. Consequently, surgery has not been recommended as a routine approach for treating SCLC for decades. However, in the past ten years, numerous retrospective analyses based on the SEER (the Surveillance, Epidemiology, and End Results) database and NCDB (National Cancer Database) have shown that surgery benefits stage I and II SCLC. Accordingly, the NCCN (National Comprehensive Cancer Network) guidelines recommended surgical treatment for T1 and T2N0 patients [3–5, 8–12]. ESMO (European Society for Medical Oncology) also proposed similar guideline practices for SCLC in 2021 [13].

Patients with early-stage SCLC have no significant symptoms. In our group, 60% of the patients were incidentally diagnosed during physical examination. On chest CT imaging, peripheral SCLC has a significantly higher proportion of smooth nodules than non-small cell lung cancer, which may be related to faster tumor cell growth and fewer stromal cells [1, 14–16]. Therefore, paying attention to the possibility of SCLC when encountering solitary pulmonary nodules with smooth edges on a chest CT scan is necessary. Also, the differential diagnosis needs to be differentiated from sclerosing hemangioma and inflammatory pseudotumor. SCLC tends to proliferate actively. The average SUV value in this group of patients who underwent PET/CT examination was 8.8, which was higher than that of non-small cell lung cancer [17]. PET-CT also provides more accurate preoperative staging of SCLC [18, 19], which is important to make a clinical strategy.

Our results also showed that clinical early-stage SCLC (stage I-IIIA) achieved a better 5-year survival rate following surgical management. The survival analysis of this group showed that the mean OS was 44.8 months, and the 5-year survival rate was 46%, which is encouraging and consistent with the results of several previous retrospective analyses [3–5, 8–11, 20–22]. The 5-year survival rate of stage I was 52.1%, which was a favorable outcome as expected. Similarly, the very limited stage (T1-2N0M0) also obtained solid benefits from oncological resection, according to several retrospective studies [4, 8, 21, 23]. So, small peripheral tumors clinically staged as N0 are recommended for surgical resection rather than needle aspiration biopsy to exclude SCLC. Patients with stage IIIA SCLC were also found to have improved OS in the present study, which is consistent with a propensity score matching study reported by Gao et al.[22]. The results indicate that patients with selected clinical stage IIIA SCLC whose lymph nodes could be completely dissected could be recommended for indications of surgery. In this cohort, there were seven cases with advanced stage, including five with IIIB and 2 with IV. The survival rate of the advanced stage was very poor, and the 5-year survival rate was zero even though these cases were treated with surgical resection and systemic chemoradiotherapy. The 5-year survival rate in the chemoradiotherapy group was better than that previously reported [21, 24], which might be due to the higher percentage of patients in the early stage of this study.

Lobectomy is still the preferred surgical approach for early-stage SCLC, and we found a significant difference in OS between the lobectomy group and the sublobar resection and pneumonectomy groups, which is also consistent with the results of previous studies [3, 22, 25]. In addition, although the incidence of mediastinal lymph node involvement in SCLC was high, the R0 resection rate in this group was still as high as 97%, which is related to strict preoperative evaluation.

In addition to surgical approach, age, surgical method, TNM stage, N stage, and vascular tumor thrombus were associated with survival time. Among them, multivariate analysis revealed lymph node involvement to as an independent survival risk factor. Therefore, preoperative lymph node biopsy should be considered when mediastinal lymph node involvement is suspected in preoperative imaging evaluation.

All incidentally confirmed SCLC should receive systemic adjuvant therapy after surgery [26]. Although no survival benefit was observed following postoperative radiotherapy in this group, mediastinal radiotherapy and chemotherapy were recommended in patients with lymph node involvement [27, 28]. The lack of observable benefits from radiotherapy may be related to the low number of cases. Previous retrospective analyses also found no survival benefit from postoperative radiotherapy (including prophylactic whole cranial irradiation) or thoracic radiotherapy following postoperative chemotherapy [29]. Prospective studies or larger retrospective analyses are expected to confirm this aspect in the future.

Recently, immune checkpoint inhibitors have been found to improve outcomes in patients with extensive-stage small-cell lung cancer in several randomized controlled trials (RCTs) [30, 31]. Anti–programmed cell death 1 (PD1) and anti-programmed death ligand 1 (PD-L1) monoclonal antibody checkpoint inhibitors are increasingly used in the adjuvant therapy of small cell lung cancer. Several RCTs have reported that antiangiogenic agents such as bevacizumab, an anti-VEGF monoclonal antibody, also led to improved PFS in small-cell lung cancer [32, 33]. Accordingly, immunotherapy and vascular targeted therapy were combined with traditional chemotherapy in 17 patients in two groups; however, they still cannot be considered standard care for SCLC.

The present study has several limitations. First of all, this was a retrospective analysis, with the relatively small number of cases, the large time span, the different characteristics of patients, and high heterogeneous postoperative treatment. Second, the study did not include patients with limited-stage SCLC without surgery as control during the same period. The subjects were patients with SCLC who were incidentally discovered after surgery, and the indications were different from those patients with SCLC confirmed by pathology before the operation. Also, neoadjuvant chemotherapy was not added. Neoadjuvant chemotherapy combined with surgery may be a reasonable option for clinical N2-stage patients [34]. Despite these several limitations, the present study reflects the actual clinical outcomes of postoperative unsuspected SCLC patients.

In conclusion, this study shows that surgery and adjuvant therapy have encouraging outcomes in postoperative unsuspected SCLC. Patients with early-stage SCLC, including stage II and IIIA, can benefit from surgery, and standard lobectomy plus systematic lymph node dissection is recommended. Systemic therapy should be performed postoperatively. Future randomized controlled prospective studies are needed to confirm whether preoperative neoadjuvant chemotherapy or neoadjuvant chemotherapy plus immunotherapy can further prolong survival time and whether to perform thoracic radiotherapy after surgery.

## Data Availability

The datasets used and/or analyzed during the current study are available from the corresponding author on reasonable request.
